# Congestion of Left Lung

**Published:** 1853-09

**Authors:** D. L. M’Gugin

**Affiliations:** Professor of Physiology, Pathology and Clinical Medicine in the Medical Department of the Iowa University


### IOWA MEDICAL JOURNAL.

VOL. I. KEOKUK, IOWA, SEPTEMBER, 1853. NO. 2.

### ORIGINAL COMMUNICATIONS.

### *Congestion of the Left Lung.*

BY D. L. M'GUGIN, M. D.

Professor of Physiology, Pathology and Clinical Medicine in the Medical Department of the Iowa University.

During the early part of the month of April last, I was called to visit, at one of the public houses of this City, Mr. ——— a young man residing some distance in the interior of the State. He came to the city with a team, laden with produce for market, and on the way suffered much from exposure to rain and cold. On the way he was attacked with rigors, followed with pain in the left side of chest, cough, pain in the head and great prostration. Aided by others, he was able to reach this place two days after the occurrence of the above mentioned symptoms.

I found him physically prostrated and mentally depressed. There was cough, with sputa frothy and albuminous, pain in the left side, hurried breathing, lividity of the lips, and a dark livid circle under the eyes, great deficiency of the capillary circulation, pulse 95 and small, tongue furred, diminished resonance over left lung, crepitant rale, particularly at the close of the inspiratory, and at the beginning of the expiratory movements. This was augmented by deep and full breathing. In this effort the thoracic muscles refused freely to expand, particularly those of the left side. An increased duty was imposed therefore upon the diaphragm and the abdominal respiratory muscles. The appetite had failed, the stomach being irritable but the bowels were regular. Diagnosed as at the head of this article. The great loss of the capillary circulation and the great depression of the system, indicated the use of stimulants, internally and externally, with the use of rubefacients over the chest opposite the left lung. Carb. ammonia, camph., opium in small doses, and warm wine were alternately given and ol. turpentine with vinegar, mustard, &c., were used externally, with pediluvia and warm sand in bags to the feet.

This course was vigorously pursued and the remedies changed, or combined, so as to ensure their most potent influence, for 10 hours, with but little or no benefit. Indeed, in many respects the symptoms were aggravated. The pulse remained feeble and depressed, the countenance wore a most anxious expression, the lips were even more livid, the circle under the eye darker, the skin wore a decidedly cadaverous aspect, the larger veins were shrunken and with their intensely dark blue color, furnished a most unnatural contrast with the bloodless and deathly color of the skin, the breathing more hurried, difficult, and distressing, slight mental aberration and the extremities still cold.

Such were the symptoms and their recital will convey to the intelligent reader the precarious condition of the patient. Here lay a young man of athletic frame, of fine physical proportions, in the vigor of youth, fast sinking under his malady, the force of which was hourly accumulating, and unless something more decided was done, he must inevitably sink. What that something should be, was a source of much doubt and anxiety. A deep solicitude begat enquiry, and set the thoughts actively in motion. Here was a case manifestly of congestion of the left lung. There was also a general diseased condition with doubtless a hepatic obstruction; there was great feebleness of the heart's action, which the stimulants failed to awaken to full activity and tonicity, a deficient capillary circulation which the warmth, stimulants, excitants, and rubefacients were incompetent to arouse, and it was plainly evident that in proportion to its decline, in that proportion the special congestion was augmented, and it was also as clear that the mobility of the blood through the veins and capillaries of the lung, diminished in the ratio of the special accumulation.

Under the weight of this, the dynamia was fast failing, to sustain which all our efforts had been unavailingly exerted; indeed, instead of accomplishing good, the difficulty was apparently aggravated by it. The lung could not be aroused to throw off its load or disburden itself, and the persuasions used only tended to annoy and aggravate it the more.

Obviously there was hepatic obstruction, which of itself often favors pulmonary accumulation and congestion. This, conjoined to the long exposure to the keen winds of the prairies, during a tedious and protracted journey, had forced away from the skin its normal share of blood and by a retropulsive force had driven it upon the lung. Some local irritation may have remained after the apparent subsidence of a catarrh under which he had labored, and united to the lung during the action of those causes which gave a centripetal direction to the fluids. That there was overdistention there was no doubt, that atony of the vessels resulted from the embarrassing pressure of the accumulation, was just as reasonable a supposition.

Such were the reflections indulged in whilst regarding this case, the pathology of which was more easily reconciled than the *modus medendi* for relief. If this condition continue, he will sink because the vital effort is not equal to the removal of the threatening accumulation, and if it should prove competent to this task, there will follow reaction with inflammation, exudation, or abscess may form, to say nothing of the present danger of hemorrhage. And as these thoughts were revolving in the mind, I silently hoped that a slight hemorrhage would occur from that lung, in order that it might diminish the engorgement; and yet this was not without its dangers, for a trifling excess might divide the slender fibre which tied him to life. I had neglected to say that cupping was resorted to, but although deep incisions were made, we were not successful owing to the impoverished capillary circulation.

I now determined to resort to venesection, maugre all the appearances and symptoms which seem to protest against it. I was convinced that to remove the great barrier in the way to the full exercise of the recuperative efforts, afforded the only prospect of success, and I was also assured that the condition of the pulse and all the evidences of vital expenditure, were so many warnings against its use generally. The failure of the stimulants to accomplish good, which seemed to be imperiously demanded, would point to the adoption of an opposite course, and I determined to pursue it under the strictest vigilance and care.

A large orifice was therefore made, and when four ounces were drawn the orifice was closed. The pulse was examined and found slower and somewhat more full. There was also some improvement in breathing. Time was allowed to note the permanency of the improvement, and when the result continued favorable, the arm was again ligated and as much more drawn. The blood improved in color as well as in consistency. The other symptoms were also noted. The pulse lost much of its frequency and acquired more volume. The breathing, still too much hurried and labored, was nevertheless much more free, less painful and the rigidity of the thoracic muscles nearly overcome. The face brightened and the relief experienced, brought back hope and the expression of his countenance betrayed the pleasing mental emotion. Pleased with the result and astonished at the effect so soon accomplished, I watched the symptcms closely. Very soon the veins in the face became full, and upon examining those upon the hand and elsewhere they also were found full; the skin became warm, so much so that the patient complained of it; was accompanied by a tingling over the surface, which in ten minutes gave way to a free and natural diaphoresis. There was now an abatement of all the symptoms but not an entire relief from the pain in the side and the cough. A blister as a derivative and to favor the centrifugal tendency was applied to the side. The following was also given:

℞ Acetas morphia grs. ij.
Ipecac pulv. grs. viij.
Sub. mur. Hyd grs. xij.
Gum camphor pulv. grs. x.
M. Divid in chart. no. viij.

One of these every four hours.

Next morning, 12 hours after, the V. S. much improved. Had rested well. Breathing nearly normal. Pulse 85, soft and more full. Crepitant rale subsiding. Skin warm and moist. Tongue less loaded. Cough almost entirely gone. Spurta muco-purulent and free.—No pain upon full dilatation of the chest by deep inspiration. Slight dullness in subscapular region, the walls of the chest expanded freely, equally and naturally, the diaphragm and abdominal muscles doing no more than their legitimate work in the respiratory act, the countenance assumed a normal expression, and the lips and skin had lost their lividity, indicating that there was now a more perfect circulation of the blood. The blister had vesicated freely, and because the skin had been previously excited by the mustard, the bleb produced was full of lymph, standing as a jelly, after the cuticle had been punctured and to some extent removed.

He continued to improve and in a few days left for his home.

This case in its features differs nothing from many other cases whose history is still fresh in the minds of those who may read this article.—The practice however, is different from that which I have myself pursued in former years. Induced to expect reaction and an accompanying rise in the pulse, stimulants have been persisted in until the vital forces had sunk so low as to be beyond the possibility of resuscitation. It is indeed a nice point to determine, because you have an admonitory hand raised against any and all measures looking to a further extinction of the powers of the system. The warnings in this case were most emphatic, and when we chose to despise and contemn them, we were assuming a fearful responsibility. Sufficient time had elapsed, and every effort had been exerted to bring about reaction, and still as no good was accomplished, it was high time to make some desperate effort to arrest the progress of his downward course. There was a cause to be removed ere this could be obtained, and this being accomplished, it would place him in the most favorable position for a final escape.

Had reaction been induced, we would have had the currents of blood set in motion, whose onward tide would have swept away the accumulated masses in the veins and capillaries of the lungs, the walls of which being distended by the presence and the force of the accumulation, an atony or loss of tonicity of these vessels obtained, the blood therefore becomes stagnant or was arrested in them to a great degree? Constitutional symptoms would, under such circumstances, be reasonably expected, because the blood, being impure from imperfect æration, would, when it would be languidly forced into the systemic circulation, produce a depraved condition of the whole system, and if it be removed, grave results would obtain with perhaps a fatal termination.

This case has been reported, to show the imperative demand for bloodletting in the early stages of pneumonia; and that when congestion takes place to an exalted degree, the patient may die from loss of the dynamic forces, and also that when active stimulation fails after a fair trial, it not only proves the necessity for an opposite course, particularly bloodletting; but that this is demanded because it relieves the organ on which the accumulat'on obtained by setting the waves of blood in motion.

The case has its interest in another particular namely, that the crepitant rale indicated congestion; that this being most evident at the close of the *inspiratory* and the beginning of *expiratory* movements showed the difficulty to be general and deep; that there could have been no solidification, because of the sudden improvement after bleeding and the immediate return to nearly normal resonance, with a complete amendment of the pulmonic and all other untoward symptoms.

